# Evaluation of Vitamin-Based Biostimulants for Disease Suppression and Sustainable Almond Production Under Commercial Orchard Conditions

**DOI:** 10.3390/plants15172606

**Published:** 2026-08-26

**Authors:** Manjula Nishantha Udagepolage Don, Singarayer Florentine, Chris Turville, Kithsiri Dassanayake

**Affiliations:** 1 The Future Regions Research Centre, Institute of Innovation, Science and Sustainability, Federation University Australia, Mount Helen, Ballarat, VIC 3350, Australia; s.florentine@federation.edu.au (S.F.); c.turville@federation.edu.au (C.T.); 2 Applied Chemistry and Environmental Science School of Science, STEM College, RMIT University, 124 La Trobe St, Melbourne, VIC 3000, Australia; 3 Ag Analytics, 540 Swanston Street, Carlton, Melbourne, VIC 3053, Australia; kbd@aganalytics.com.au

**Keywords:** prunus dulcis, vitamin B complex, induced systemic resistance, biostimulants, integrated disease management, foliar vitamin application, pesticide residue reduction, sustainable agriculture, almond hull utilisation, plant immunity

## Abstract

Almond (Prunus dulcis) production generates large quantities of hull by-products, the safe utilisation of which can be affected by pesticide residues resulting from conventional disease management practices. Identifying practical approaches that reduce pesticide use while maintaining crop health is therefore an important step towards more sustainable almond production. This study evaluated vitamin-based biostimulants as a potential alternative disease management strategy under commercial orchard conditions. Field experiments were conducted over two consecutive growing seasons in commercial almond orchards in Australia. Eight vitamin formulations containing vitamin B complex, vitamin B_1_, vitamin C and vitamin E were evaluated for their ability to reduce disease incidence in leaves and whole nuts, together with their effects on chlorophyll content, flower-to-nut conversion, kernel nutrient composition and other production-related parameters. A formulation containing 50 µg mL^−1^ vitamin B complex together with vitamin B_1_ consistently reduced disease incidence to levels comparable with the grower standard chemical programme. Selected vitamin treatments also maintained chlorophyll content, kernel nutrient composition and flower-to-nut conversion without adversely affecting crop performance. Overall, the results indicate that vitamin-based biostimulants have the potential to become a practical component of integrated disease management in commercial almond production. Their use may contribute to reducing reliance on conventional pesticides while supporting the sustainable utilisation of almond hull by-products and improving the environmental sustainability of almond production systems.

## 1. Introduction

Almonds (*Prunus dulcis*) stand out as one of the leading nut crops worldwide. In 2025, the total global almond production of the top five producing countries—the United States, Australia, Spain, Türkiye, and Morocco—reached 2.83 million tons, with the United States contributing over 70% of this total [1]. Australia is currently the second-largest almond producer globally, accounting for over 8% of the world’s production. The growth of the Australian almond industry has been significant, with its planted area expanding from 8102 hectares in 2007 to 58,579 hectares in 2023 [2].

In 2020, Australia harvested 114,427 tons of almond kernels, yielding a farm gate value of AU$545 million and contributing AU$1.63 billion to GDP [3], which makes the almond industry vital to the national economy. A key challenge for the almond industry’s sustainability, however, lies in its ability to identify environmentally sound ways to dispose of its post-harvest by-products. The main by-products of almonds are hulls and shells, with hulls making up 54% of the entire nut and shells 19% (that is, 70–74% of the entire nut). The edible almond kernel, by contrast, constitutes only 27–30% of the total nut [4]. By 2023, the Australian almond industry produced over 258,000 tons of almond hulls and shells.

Almond hulls and shells can serve many purposes, such as livestock bedding, dairy feed, and energy sources [5]. Almond hulls are especially valuable as a feed option that improves digestibility and increases milk fat percentage in lactating cows, potentially constituting up to 20% of their diet [6]; they can also be useful as a source of dietary energy and fibre in poultry diets [7]. As the primary uses of almond hulls in Australia and in most important almond-producing regions around the world are for animal feed and bioenergy generation [8], a major concern is how the chemical pesticides used to manage pests and diseases on almond farms can lead to pesticide residues in the almond hulls. In fact, using almond waste products on farms has been discouraged due to the risk of disease spread [9]. To deal with the challenges related to the safe use of almond hulls in livestock feeding, both state and federal regulators in Australia have established mandatory (that is, legally enforceable) industry standards for almond and other products, which are known as maximum residue levels (MRLs) and are based on the pesticide risk profiling of animal feeds [10]. The aim of these regulations is to address hazards related to chemical pesticide residues in almond hulls used as livestock feed [11]. The challenge for the almond industry is how to continue to dispose of almond hulls through livestock feeding but at the same time reduce chemical pesticide usage while maintaining crop health and productivity.

In this study, we use an external application of vitamins to enhance almonds’ internal resilience and to protect them against the most common pests and diseases. Several previous studies have investigated the roles of vitamin B1 (thiamine) and vitamin B complex in activating plant disease resistance mechanisms in some crop species [12]. There are eight types of vitamins that make up vitamin B complex: thiamine (B1), riboflavin (B2), niacin (B3), pantothenic acid (B5), pyridoxine (B6), biotin (B7), folate (or ‘folic acid’) (B9) and cyanocobalamin (B12) [13]. Vitamin B1 (thiamine) has been shown to build biotic and abiotic resistance in plants [14], and a study by Ahn et al. (2005) found that treating Arabidopsis plants and vegetable crops with vitamin B1 made them resistant to bacterial, fungal and viral infections [15,16]. Vitamin B1 has the ability to induce plants’ systemic acquired resistance (SAR) [16], which is a type of induced resistance activated in plants after exposure to pathogenic or non-pathogenic microbes or artificial stimuli such as the chemical, salicylic acid. Salicylic acid is an immobile signalling agent that has the ability to induce pathogenic-related (PR) genes (Gupta, 2014), such that when a plant is attacked by a pathogen for a second time, it responds more quickly and efficiently because the SAR can access past memory of the attack [17]. In addition to vitamin B1 and vitamin B complex, vitamin E has also been found to improve resistance against plant diseases [18], while vitamin C has a demonstrated ability to control pear disease and improve the quality of the fruit. Many other studies have also shown how vitamins can be used to control pests and diseases [19].

To date, only a few studies have evaluated the effectiveness of vitamin B complex or any other vitamins (such as vitamins C and E) in almond production to provide an alternative to chemical control or as a part of Integrated Pest Management (IPM) practice. The aim of our research has been to assess the effectiveness of multiple new formulations of vitamin B complex, as well as vitamins C and E, against three common fungal diseases found in almond orchards: rust (*Tranzschelia discolour*), bacterial spot (*Xanthomonas arboricola pv. pruni*) and shot-hole (*Wilsonomyces carpophilus*) [20]. Anthracnose—*Colletotrichum gloeosporioides*—and hull rot—*Rhizopus spp.* or *Monilinia spp*—are the other two main fungal diseases that impact almonds [20]. While most almond growers are currently able to control these diseases with chemical fungicides, which keeps the disease severity below economic threshold levels [21], diseases such as hull rot can lead to complexities such as an increase in the occurrence of lower limb dieback (LLD) [22]. Unfortunately, chemical pesticides can also leave chemical residues that must be eliminated if the almond hulls are to be used as an economic commodity [23].

This study investigates the transformative effects of various vitamin formulations on the internal immunity of almond plants and compares these effects to those derived from the standard chemical treatments used by almond growers. Specifically, we analyse vital parameters related to almond production to understand whether the use of exogenous vitamins enhances—rather than hinders—the yield and quality of almond kernels without the need for potentially harmful chemicals. The objective, therefore, is to test the validity of our hypothesis and practically provide a solution for the sustainable future of almond agriculture in Australia and globally. Through this forward-thinking approach, we aspire to lead the almond industry towards greater sustainability by reducing chemical contamination in almond hulls and opening up new opportunities for their use.

## 2. Methodology

### 2.1. Site Selection and Research Design

**Figure 1:**
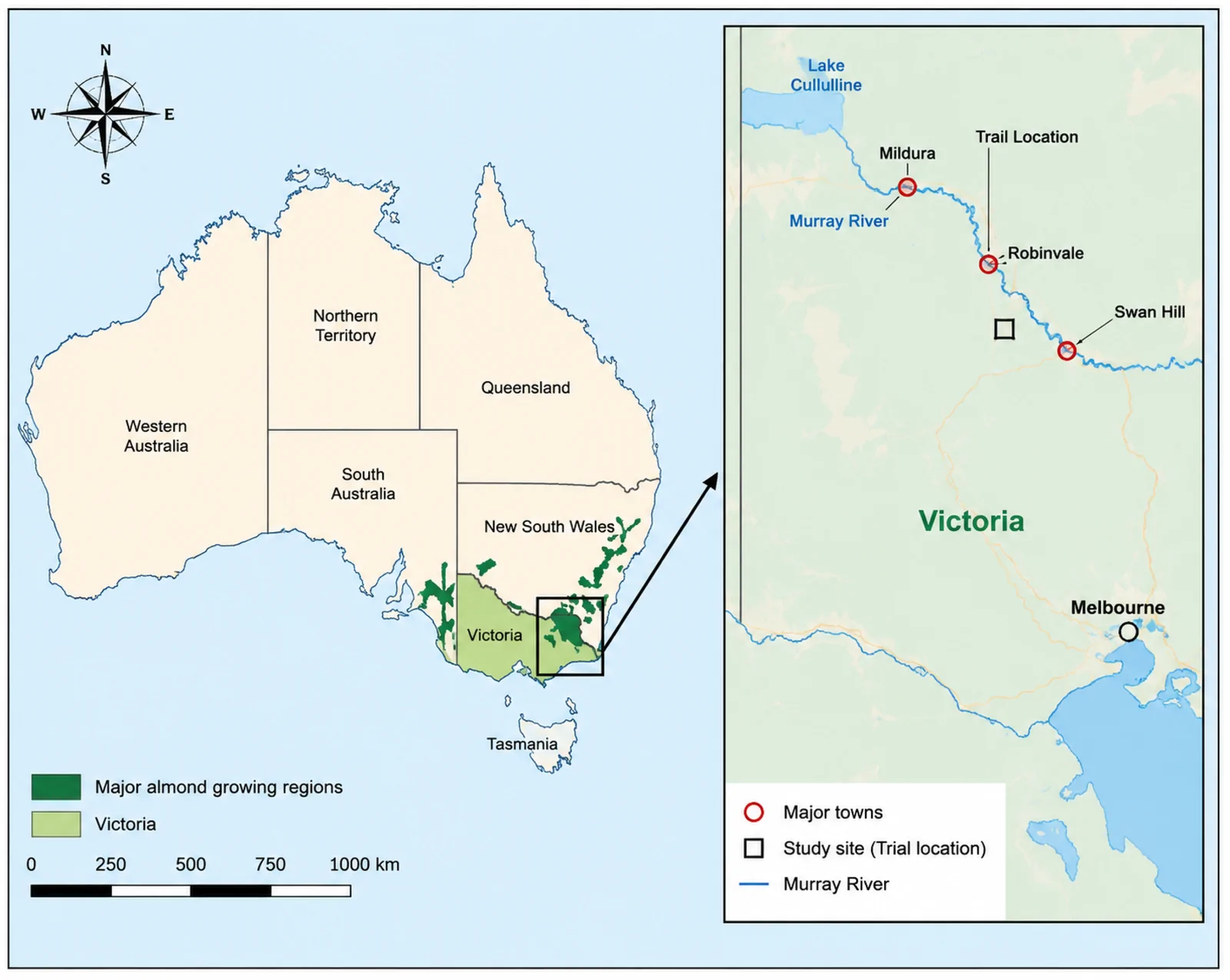
Location of the experimental site in the major almond-growing region of northwestern Victoria, Australia.

The main almond-growing region in Australia is in the northwest of the state of Victoria, located between the city of Swan Hill and the town of Robinvale, and the sites for this study were selected from almond farms in this region. Figure 1 shows the location of the experimental almond orchard in northwestern Victoria, Australia, showing the study site within the principal almond-growing region. This study was conducted over three years, from 2021 to 2024, to cover two complete consecutive growing seasons with repetitive data.

This research was conducted on 66 rows of almond trees, each comprising 138 almond trees distributed across two neighbouring plots. According to the randomised design, we have identified 72 treated replicates (across the three varieties), 12 control replicates (across the three varieties), and 12 grower standard replicates (across the three varieties).

**Figure 2:**
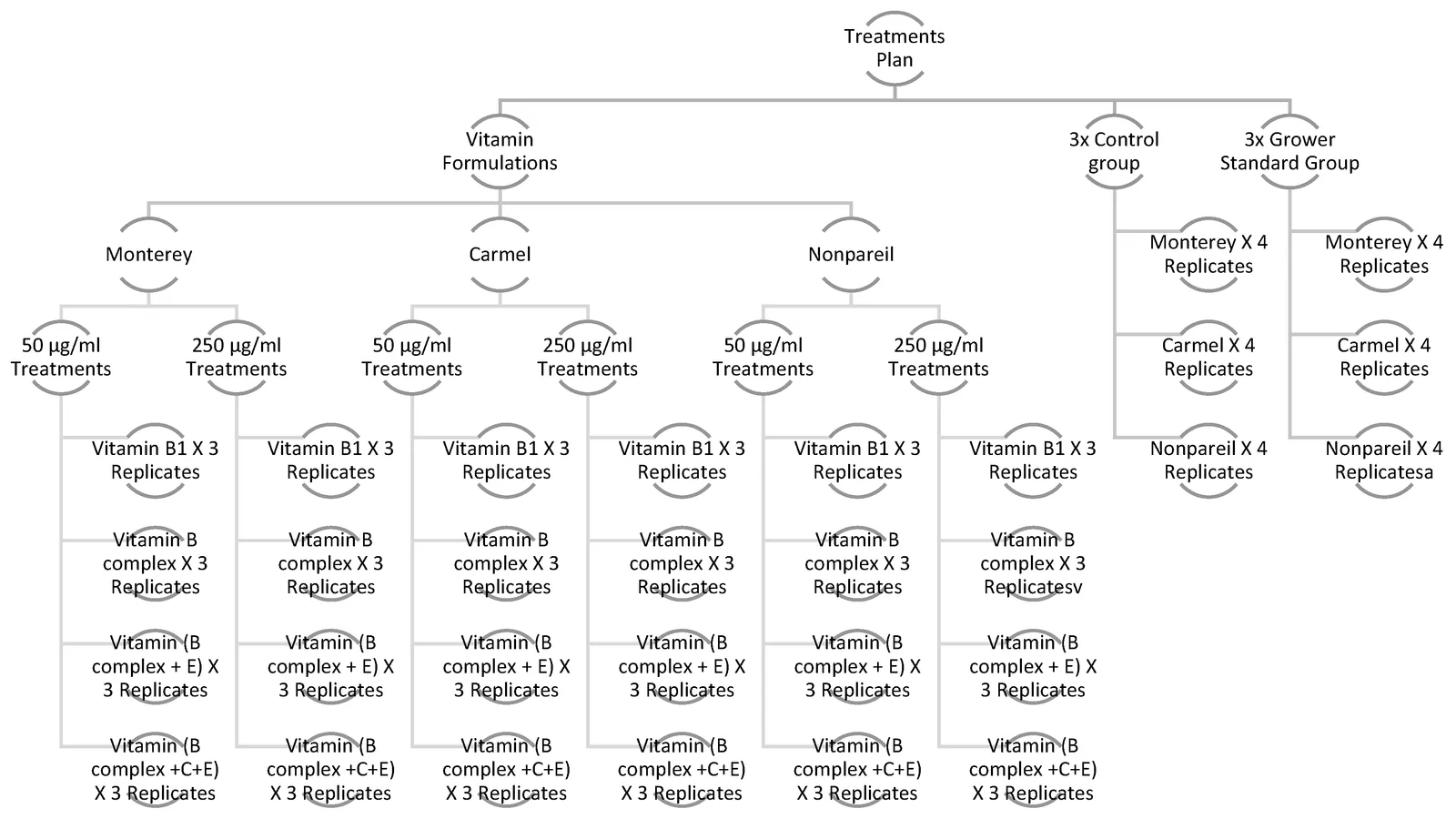
Treatment plan.

In our study, four different formulations of vitamins B, C and E were utilised, with each formula prepared at two different doses. Five percent of plant-derived amino acids and 5% of biologically active organic molecules (BAOMs) were incorporated into each of these formulations (treatments) to enhance absorption efficiency. To optimise the activity of these vitamin treatments, no additional spray adjuvant was employed. Those plots (five tree groups that included two buffer trees) treated with each grower’s standard chemical were taken to be the designated grower standard. This study employed a randomised complete block design (RCBD) where three different almond cultivars were selected from three adjacent rows, with three plants chosen for each treatment and three replicates of each treatment. Treatments were applied once during the nut development stage and once during the nut maturation stage, and each treatment was applied with a water rate of 1250 L/Ha, which is standard across the almond industry, to ensure adequate coverage for producing almonds. As shown in Figure 2, the treatment plan included 72 treated replicates, 12 control replicates and 12 grower standard replicates.

### 2.2. Treatments and Application Rates

Each replicate comprised three groups of 3 almond trees arranged for commercial production. Replicates were separated by a buffer zone that consisted of either a two-to-three-row spacing (approximately 12 m) or a buffer area (approximately 12 m) designed to minimise cross-contamination during spraying. Foliar spraying was carried out using a motorised 12v spray unit, and 5 litres of tank mix was applied to each plant until fully covered, to the point of drip.

The commercial vitamin B complex product used in the field trial contained the B vitamin group described in the Introduction (B1, B2, B3, B5, B6, B7, B9 and B12). The experimental records identify the field application rate of the formulated B complex product but do not provide verified per-component concentrations for each individual B vitamin. Accordingly, the manuscript reports the applied mass concentration of the commercial formulation and does not infer unrecorded individual-vitamin concentrations.

Table 1 consolidates the treatment formulations, application concentrations and abbreviations used throughout the manuscript. The vitamin treatments are grouped by application concentration (50 and 250 µg mL^−1^) to improve readability.

### 2.3. Grower Standard Chemical Programme

The grower standard treatment represented the commercial disease management programme routinely implemented by the participating orchard throughout this study. This programme consisted of the fungicide and insecticide applications normally applied by the grower according to seasonal disease pressure, pest monitoring and commercial management practices. The grower standard plots received all pesticide applications undertaken by the orchard manager during the experimental period, whereas the untreated control received neither vitamin formulations nor grower standard pesticide applications. The vitamin-treated plots received the experimental vitamin formulations in place of the grower standard foliar disease management treatments, while all other orchard management practices, including irrigation, fertilisation and general cultural operations, were maintained uniformly across all treatments.

### 2.4. Data Collection and Evaluation Methods

A systematic evaluation of pest damage was conducted to comprehensively assess the impact of treatments, and this included inspections of leaves and kernels to identify the presence of damage. The assessments included the Visible Disease Assessment (VDA) of the almond leaves, a total fungal damage assessment of the almond nuts (including hull rot), and a pest damage assessment of the almond nuts, which covered issues such as Carpophilus beetle infestation, carob moth damage, and kernel gumming and aborted kernels.

The sampling method used to collect almond nuts for the assessments was consistent across all studies. Each sample contained 20 mature almond nuts harvested from each replicate one week prior to the official harvesting period. These nuts were collected from shoulder height around each tree to ensure comprehensive coverage, and each treatment included three replicates for each of the three varieties (Monterey, Carmel and Nonpareil). The method used for leaf sampling was similar to that used for the nut collection: 20 mature almond leaves were gathered from each replicate at designated times for analysis, and these were also collected from shoulder height around each tree.

The collected nut samples underwent various analyses, including evaluations of fungal damage and pest damage, weighing the oven-dried nuts, nutrient analysis, and maximum residue limit (MRL) testing. The leaf samples were analysed to assess leaf pest and disease damage, nutrient content, and chlorophyll levels.

For disease analysis to understand the impact of the vitamin treatments, 96 leaf sample groups (20 leaves per sample) were analysed. Infected leaf counts were conducted on almond trees during growing seasons 1 and 2. Treatment-level disease data are presented in the Results, while the detailed linear mixed model output is provided in the Appendix A. The two seasons were analysed separately due to differing weather conditions and variations in disease pressure. Control trees received the grower standard chemical foliar applications, while the treated trees received the developed vitamin formulations. This assessment was conducted with regard to the number of damaged visual sites on leaf surfaces, consisting of 20 leaves in each sample.

After the nut samples were collected, each sample was placed in its own sealed plastic bag, which created the high-humidity conditions that are ideal for fostering fungal growth, and the sample was incubated for 14 days to assess post-fungal disease development. The samples were kept at a temperature of 4.0 °C and shielded from direct sunlight. The rest of the nut samples were manually cracked open using an almond nutcracker to separate the hulls from the kernels, both of which were then sent to an independent lab, Analytical Laboratories and Technical Services Australia Pty Ltd. (ALTSA), for nutritional and maximum residue level (MRL) analysis. Nutritional analysis was conducted on oven-dried almond kernel and leaf samples that were finely ground and subjected to wet-acid digestion in accordance with the Western Region reference methods for plant tissue analysis (WREP-125) and the ALTSA standard operating procedure for wet-acid digestion (SP.SOP.006). The digested samples were analysed for macro- and micronutrients by utilising Inductively Coupled Plasma–Optical Emission Spectrometry (ICP-OES) and Inductively Coupled Plasma–Mass Spectrometry (ICP-MS), following U.S. EPA Methods 6010C, 6020B, and 3051A, as well as ALTSA accredited laboratory procedures (SP.SOP.016 and SP.SOP.017), as appropriate for each element. MRL analysis was conducted via multi-residue pesticide scanning, combining QuEChERS sample prep, liquid chromatography–tandem mass spectrometry (LC-MS/MS), and gas chromatography–mass spectrometry (GC-MS/MS). To conduct a yield analysis, hull and kernel samples were placed in separate labelled paper bags and dried in a forced-air oven at 70 °C until each sample achieved a constant weight [24]. The kernel and hull samples were then weighed separately, and a statistical evaluation was conducted.

Chlorophyll levels were measured using the SPAD (Soil Plant Analysis Development)-502 Plus, a well-established field instrument in the almond industry. This portable and non-invasive device was specifically designed to evaluate leaf chlorophyll content. The device measures the absorbance of leaves in two wavelength ranges, blue (400–500 nm) and red (600–700 nm), and shows no absorbance in the near-infrared spectrum. By utilising these metrics, the instrument computes a numerical SPAD value that correlates with the chlorophyll amount found in the leaf [25].

The flower-to-nut conversion ratio was also calculated per replicate. This ratio determines how many flowers are successfully converted into harvestable nuts and serves as a key indicator of yield performance. It is crucial that the conversion ratio does not differ significantly among treatments, as this would indicate that treatments do not adversely influence this outcome.

Statistical data analysis was carried out using IBM SPSS versions 29.0.0.0 and 30.0.0.0, and it utilised a linear mixed model (LMM), which outperforms the standard linear model by effectively managing correlated data and non-constant variability. As this study deals with multiple levels of groupings [26]—i.e., 10 different treatment groupings and three different varietal groupings—the LMM has been particularly useful and has allowed for the modelling of the dataset’s means, variances and covariances. In addition, this analysis method allows the field, row and plot of 3 trees’ replicates to be modelled as random effects. It can also handle the hierarchical nature of the data, as there are trees within plots and leaves/nuts within trees. The analysis was based on a number of assumptions. The dependent variable (such as damaged nuts and disease-infected leaves, which were tested variables) was expected to show a linear relationship with fixed factors (treatments and varieties), random factors, and covariates. Fixed effects represented the mean of the dependent variable (such as disease damage on leaves), whereas random effects described its covariance structure. Each random effect was treated as independent, and separate covariance matrices were determined for each effect (although terms defined on the same random effect may be correlated). The repeated measures model regulated the covariance structure of the residuals, with the dependent variable also assumed to originate from a normal distribution. The Bonferroni correction was used in the data analysis, where multiple statistical tests were performed simultaneously and independently, to reduce the risk of false (type I error) positives [27].

## 3. Results

### 3.1. Disease Assessment of Almond Leaves

Infected leaf counts were conducted on almond trees during growing seasons 1 and 2. Treatment-level disease data are presented in the Results, while the detailed linear mixed model output is provided in the Appendix A. The total fungal counts related to actual visual damage incidents were concentrated on observing shot-hole (Wilsonomyces carpophilus), bacterial spot (Xanthomonas arboricola), and leaf rust (Tranzschelia discolor).

The treatment-level leaf disease data are presented in the main Results to make the biological response directly visible. The linear mixed model confirmed a significant treatment effect in both seasons (Season 1 and Season 2, *p* < 0.001); the detailed Type III Tests of Fixed Effects are presented as Table A1 and Table A2 in the Appendix A Section.

As shown in Table 2, leaf disease damage during Season 1 varied among treatments and almond varieties. The untreated control recorded the highest overall disease damage (20.22%), with similar levels observed in Carmel and Monterey (7.01%) and slightly lower damage in Nonpareil (6.20%). All vitamin-based treatments reduced overall leaf disease damage compared with the control. The lowest overall damage (7.01%) was observed with B1-50 and BcCE-50, representing a substantial reduction relative to the untreated control. The grower standard also showed comparatively low disease damage (8.09%). Treatment responses varied among varieties, indicating some varietal differences in the effectiveness of the vitamin-based treatments.

For Season 2 (Table 3), leaf disease damage was greatest in the untreated control, accounting for 28.81% of the total observed damage across all varieties. By contrast, B1-50 completely suppressed visible leaf disease damage, with no damage recorded in Carmel, Monterey, or Nonpareil (0.00%). All other vitamin-based treatments also showed lower overall disease damage than the control, ranging from 6.78% to 12.71%. Among these, BcCE-50 and B1-250 recorded the lowest overall damage (6.78%), followed by BcE-250 (7.63%). The grower standard recorded 11.86% of the total damage. Overall, the Season 2 results demonstrate reduced leaf disease damage across the vitamin-based treatments, with B1-50 showing the strongest response across all three almond varieties.

**Figure 3:**
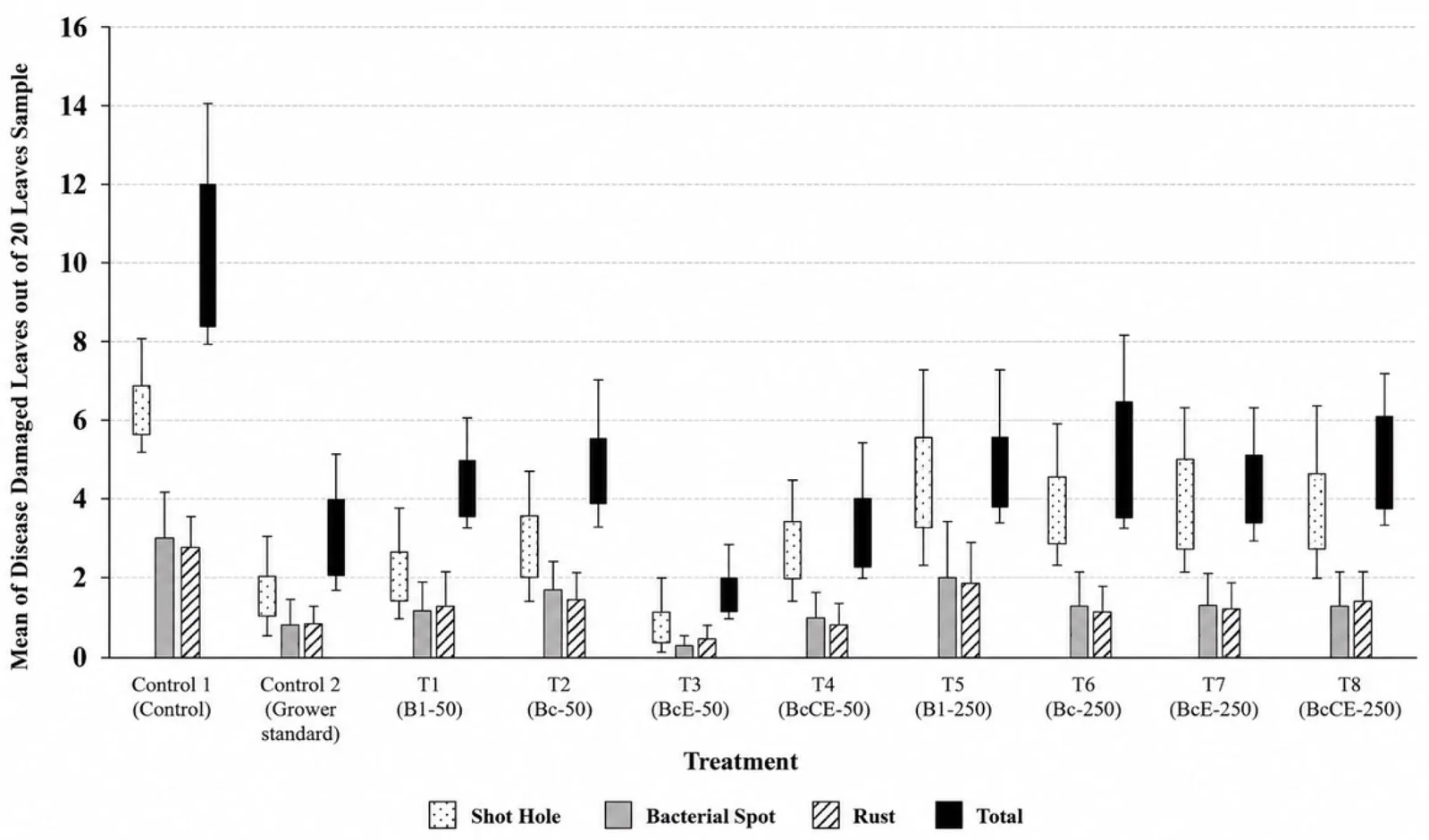
Leaf disease assessment for Season 1. Abbreviations: GS, grower standard; B1, vitamin B1; Bc, vitamin B complex; BcE, vitamin B complex + vitamin E; BcCE, vitamin B complex + vitamin C + vitamin E. Suffixes -50 and -250 denote application concentration (µg mL^−1^).

**Figure 4:**
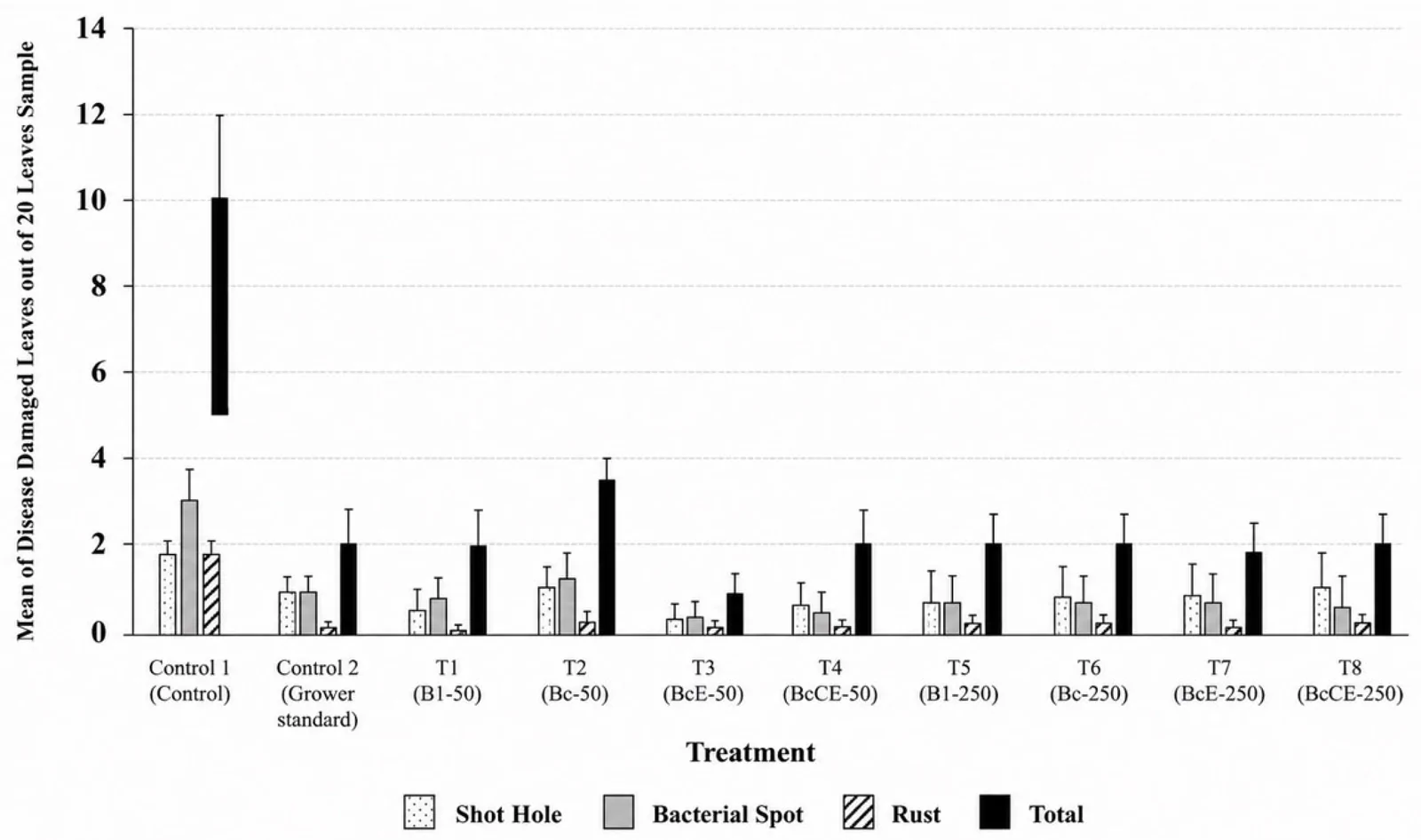
Leaf disease assessment for Season 2. Abbreviations: GS, grower standard; B1, vitamin B1; Bc, vitamin B complex; BcE, vitamin B complex + vitamin E; BcCE, vitamin B complex + vitamin C + vitamin E. Suffixes -50 and -250 denote application concentration (µg mL^−1^).

Figure 3 and Figure 4 show the mean of individual disease-damaged leaves for Seasons 1 and 2, respectively.

The X axis represents all nine treatments and the control. The total disease damage represents the sum of the mean of the shot-hole, bacterial spot and rust diseases. The Y axis represents the mean of disease-damaged leaves (from the sample of 20 leaves), excluding outliers.

The analysis of leaf damage for Season 1 (aggregating all varieties) showed a significant difference at the 95% confidence level (*p* = 0.05) between the control group (Control), which received no treatments, and the other treatments. This control group, drawn from the 20 leaf samples, exhibited the highest mean of disease-infected leaves. Notably, no significant difference was observed between the grower standard chemical control (GS) and the sample treated with vitamin treatments B1-50 to BcCE-250. Of these treatments, BcE-50 exhibited the lowest total mean damage level, which was lower than the grower standard; however, there was no statistically significant difference at 95% confidence levels.

The analysis of leaf damage for Season 2, also aggregating all varieties, revealed a pattern consistent with that observed in Season 1. There was a statistically significant difference at the 95% confidence level between the control group, which received no treatments, and the other treatment groups. The control group displayed the highest total mean of disease-infected leaves, based on the 20 leaf samples. Notably, there was no significant difference between the grower standard chemical control samples (GS) and those treated with vitamin treatments B1-50 to BcCE-250. Of these treatments, B1-50 showed the lowest mean level of total disease damage, although this was not statistically significant compared to the grower standard, while BcE-50 demonstrated the second lowest mean level of total disease damage, although this was also not significantly lower than that of the grower standard.

With respect to the impact of leaf disease damage at the varietal level, Table A3 **(in the Appendix A section)** (Season 1) and Table A4 **(in the Appendix A section)** (Season 2) present the average damage for each variety, alongside the total sum of the means as numerical values and as a percentage of the grand total. The damage for each variety has been statistically analysed but was not statistically significant. Regarding growing, Season 1 results in *p* = 0.64 and Season 2 results in *p* = 0.50.

Additionally, Season 1 experienced higher disease pressure than Season 2 in the number of incidents, 371 as compared to 118, respectively. Treatment 1 and Treatment 4 yielded the lowest sum of means of leaf damage in both seasons.

### 3.2. Disease Assessment of Almond Whole Nuts

To understand the impact of the vitamin treatments, 96 whole-nut samples, each containing 20 fully grown almond nuts, were analysed. Treatment- and variety-level data are presented in the main Results. The linear mixed model identified significant treatment effects in both seasons (Season 1, *p* < 0.001; Season 2, *p* = 0.040), while the detailed Type III Tests of Fixed Effects can be found. 

The total disease damage represents the sum of the mean (excluding outliers) of the shot-hole, bacterial spot and rust diseases. The Y axis represents the mean of disease-damaged nuts out of 20 nuts in the sample (excluding outliers). A mean line for total disease damage has been included for the sake of clarity.

Analysis of the whole-nut damage in Season 1 (combining all varieties) revealed a noteworthy distinction at the 95% confidence level between the untreated control group and the nine other treatments. The control group displayed the highest mean number of disease-infected whole nuts. Importantly, no significant difference was found between the grower standard chemical control sample (GS) and vitamin treatment samples B1-50 to BcCE-250. Of all the treated samples, the grower standard sample recorded the lowest overall mean damage level, although there was no statistically significant difference between this sample and the vitamin-treated samples at a 95% confidence level. The BcCE-50 and B1-250 treatments showed the second and third lowest mean damage levels in the whole-nut analysis.

For Season 1 (Table 4), whole-nut disease damage was the most pronounced in the untreated control, which accounted for 24.57% of the total observed damage across the three almond varieties. All vitamin-based treatments showed lower levels of nut damage than the control. Among these, BcCE-50 recorded the lowest overall damage (6.57%), followed by B1-250 (7.27%) and BcCE-250 (7.44%). The grower standard also recorded relatively low damage (7.27%). Differences were evident among varieties, with Carmel accounting for the greatest proportion of total nut damage (43.43%), followed by Monterey (36.85%) and Nonpareil (19.72%). Overall, the results indicate that the vitamin-based treatments were associated with reduced whole-nut disease damage compared with the untreated control, although the magnitude of the response varied among treatments and varieties.

For Season 2 (Table 5), whole-nut disease damage was again greatest in the untreated control, which accounted for 29.76% of the total damage recorded across all varieties. All vitamin-based treatments showed lower overall damage than the control, although the level of reduction varied among treatments and varieties. BcE-50 produced the lowest overall nut damage (1.09%), with no damage recorded in either Monterey or Nonpareil. This was followed by B1-250 (2.84%) and Bc-250 (5.69%). In comparison, the grower standard accounted for 11.60% of the total damage. A clear varietal difference was also observed, with Nonpareil contributing the greatest proportion of damage (51.86%), compared with Carmel (24.29%) and Monterey (23.85%). Overall, the Season 2 results indicate that several vitamin-based treatments were associated with substantially lower whole-nut disease damage than the untreated control, with BcE-50 showing the strongest overall response.

**Figure 5:**
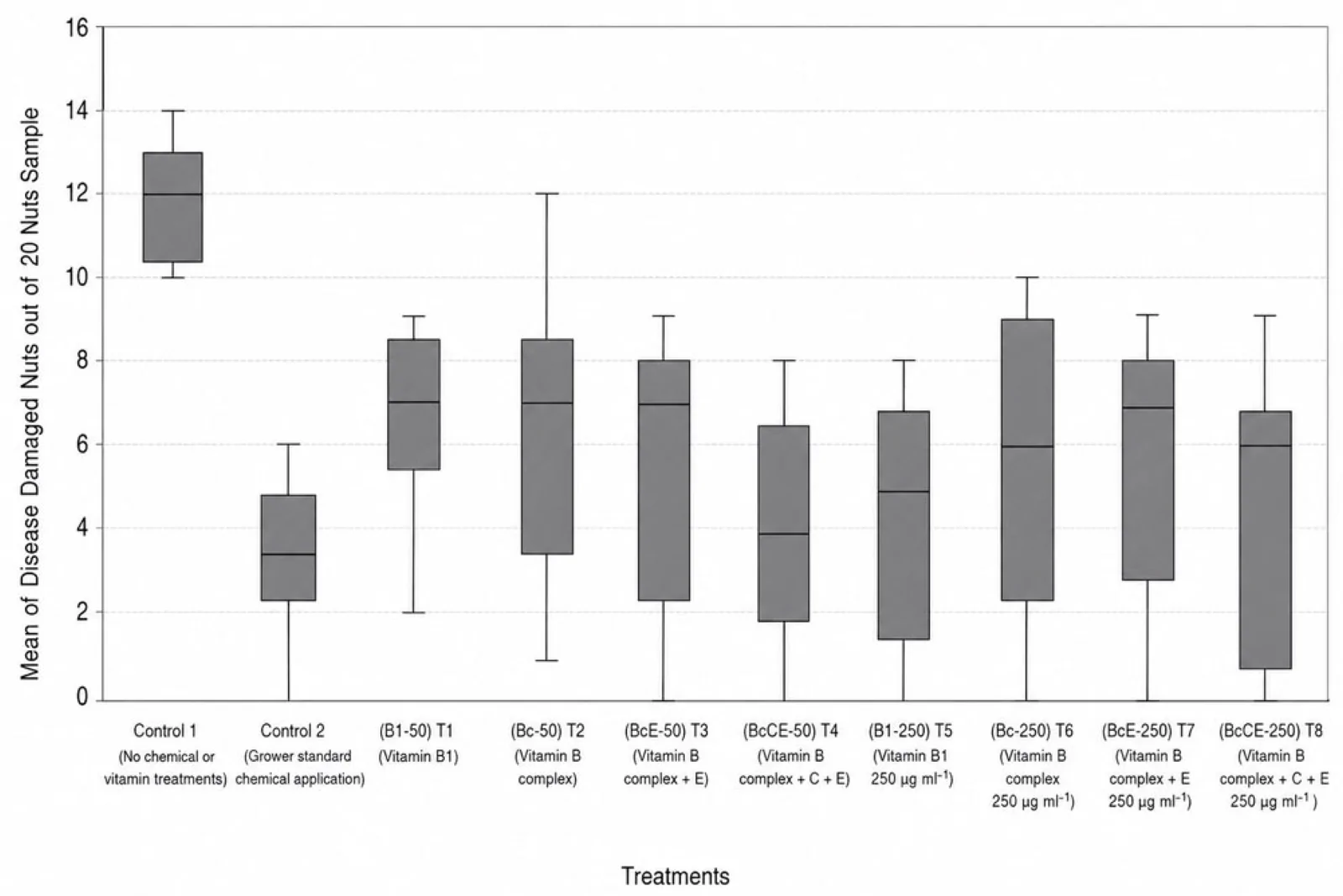
Assessment of whole-nut disease for Season one. Abbreviations: GS, grower standard; B1, vitamin B1; Bc, vitamin B complex; BcE, vitamin B complex + vitamin E; BcCE, vitamin B complex + vitamin C + vitamin E. Suffixes -50 and -250 denote application concentration (µg mL^−1^).

**Figure 6:**
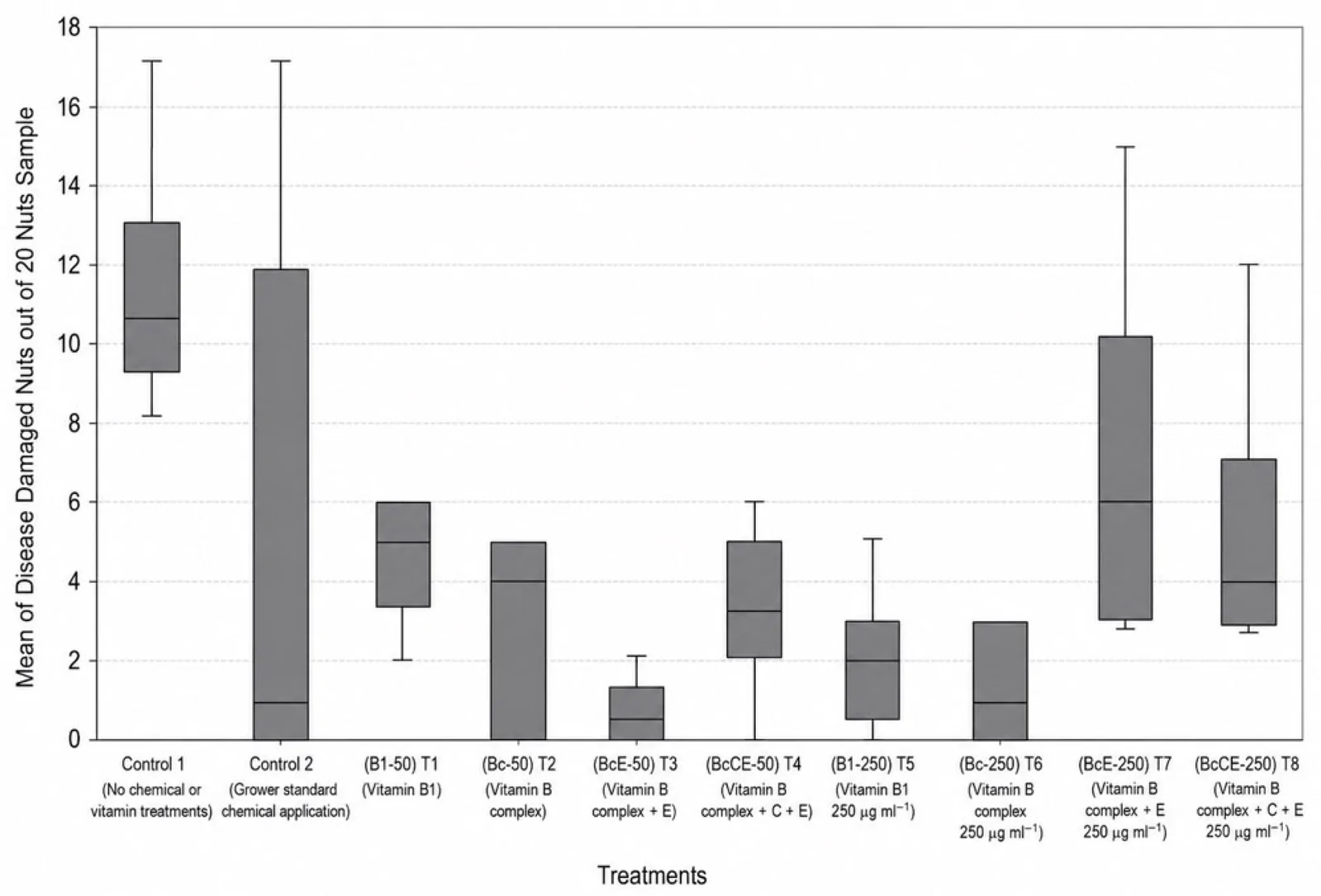
Assessment of whole-nut disease for Season two. Abbreviations: GS, grower standard; B1, vitamin B1; Bc, vitamin B complex; BcE, vitamin B complex + vitamin E; BcCE, vitamin B complex + vitamin C + vitamin E. Suffixes -50 and -250 denote application concentration (µg mL^−1^).

Figure 5 and Figure 6 show the mean of individual disease-damaged nuts in Season 1 and Season 2, respectively.

Season 2 exhibited the same statistical significance as Season 1 for all treated samples compared to the control. Furthermore, there was no significant difference between the grower standard samples and treatments B1-50 to BcCE-250. Analysis of the data from both growing seasons confirmed that all vitamin treatments significantly reduced the observed disease damage to the whole nuts. However, as with the leaf disease analysis, Season 2 showed lower disease occurrence compared to Season 1. The best whole-nut disease control was observed in BcE-50, followed by B1-250; BcE-50, BcCE-50 and B1-250 had the lowest mean disease levels across both growing seasons.

Comparing both seasons, Season 1 had higher disease pressure than Season 2 (578 and 457, respectively). In both growing seasons, B1-50 and BcCE-50 had the lowest sum of means of leaf damage. In Season 1, BcCE-50 was the treatment with the least nut damage, while BcE-50 and B1-250 had the lowest sum of means.

### 3.3. Assessment of Damaged Almond Kernels (Unproductive Kernels)

Kernel damage can arise from insect feeding or from physiological defects such as aborted and gummy nuts, all of which reduce the commercial value of the kernel. In this study, insect-related damage was attributed principally to carob moth and Carpophilus beetle, while gummy and aborted kernels were assessed separately. Overall, kernel damage was uncommon in both growing seasons. In Season 1, only one damaged kernel was recorded among 1920 assessed kernels (0.052%), whereas 19 kernels (0.989%) showed carob moth- or Carpophilus beetle-related damage in Season 2. 

Table 6 presents the almond kernel damage caused by carob moth and Carpophilus beetle during Season 1. Kernel damage was extremely low, with only one damaged kernel recorded across all treatments and varieties. This single incidence occurred in the untreated control and was associated with the Nonpareil variety. No kernel damage was detected in Carmel or Monterey, and, importantly, no damage was observed in the grower standard or any of the vitamin-based treatments (B1-50, Bc-50, BcE-50, BcCE-50, B1-250, Bc-250, BcE-250, and BcCE-250). Overall, these findings indicate negligible carob moth- and Carpophilus beetle-related kernel damage during Season 1.

Table 7 presents the almond kernel damage caused by carob moth and Carpophilus beetle during Season 2. A total of 19 damaged kernels were recorded, with the majority occurring in Nonpareil (16 kernels; 84.21%), followed by Monterey (3 kernels; 15.79%), while no damage was observed in Carmel. Among the treatments, BcE-50 recorded the highest incidence of kernel damage (10 kernels; 52.63%), followed by the untreated control (5 kernels; 26.32%). B1-250 and BcCE-250 each recorded two damaged kernels (10.53%). No damage was detected in the grower standard, B1-50, Bc-50, BcCE-50, Bc-250, or BcE-250 treatments. Overall, kernel damage during Season 2 was predominantly associated with the Nonpareil variety and was concentrated in a small number of treatments.

Kernel damage due to carob moth and Carpophilus beetle was not significant for the control versus grower standard or any of the eight vitamin treatments, which may be due to the low level of pest damage. 

Table 8 presents the almond kernel damage associated with aborting and gumming during Season 1. A total of eight damaged kernels were recorded, with Nonpareil accounting for the highest proportion (4 kernels; 50%), while Carmel and Monterey each accounted for two kernels (25%). Among the treatments, BcCE-50 recorded the highest incidence of damage (3 kernels; 37.5%). The grower standard, Bc-50, BcE-50, B1-250, and BcE-250 treatments each recorded one damaged kernel (12.5%). No damage was observed in the untreated control, B1-50, Bc-250, or BcCE-250 treatments. Overall, aborting- and gumming-related kernel damage was relatively low and occurred sporadically across varieties and treatments.

Table 9 presents the almond kernel damage associated with aborting and gumming during Season 2. A total of 31 damaged kernels were recorded, with damage relatively evenly distributed among Carmel (11 kernels; 35.48%), Monterey (10 kernels; 32.26%), and Nonpareil (10 kernels; 32.26%). Among the treatments, Bc-50 recorded the highest incidence of damage (7 kernels; 22.58%), followed by B1-50 and BcE-250, with six damaged kernels each (19.35%). The grower standard and BcCE-50 treatments each recorded three damaged kernels (9.68%), while the control, BcE-50, and BcCE-250 treatments each recorded two (6.45%). No damage was observed in the B1-250 or Bc-250 treatments. Overall, aborting- and gumming-related kernel damage was more broadly distributed across varieties and treatments in Season 2 than in Season 1.

The observed level of damage in both growing seasons was minimal. In Season 1, there were eight aborted and gummy nuts out of a total of 1920 nuts (0.416%), while in Season 2, the damage increased to 31 kernels out of 1920 (1.614%). However, these findings were not statistically significant at the 95% confidence level, and no difference was therefore observed between the untreated control, the grower standard and the vitamin-treated kernels.

### 3.4. Flower-to-Nut Conversion Ratio Assessment

**Figure 7:**
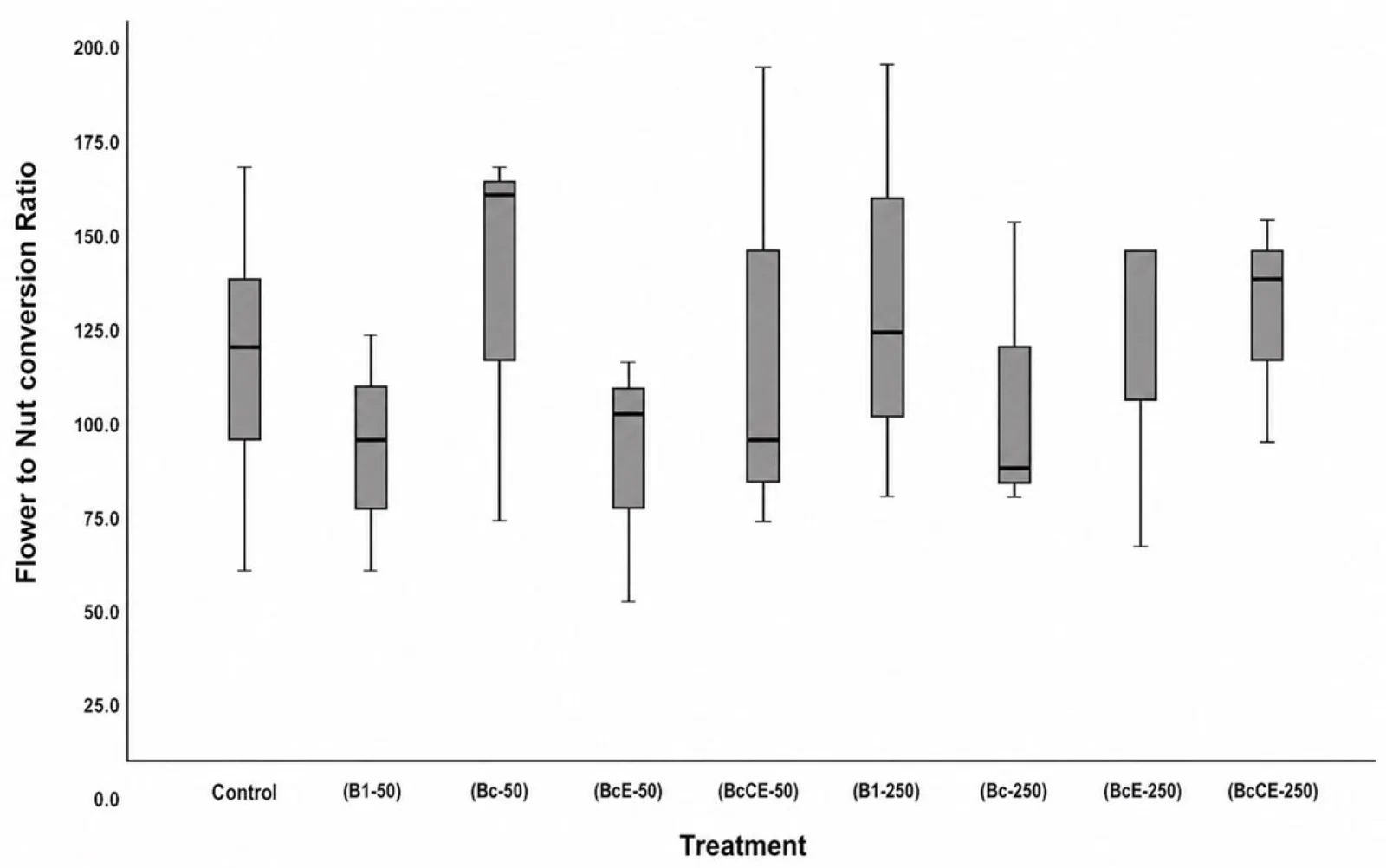
Flower-to-nut conversion ratio analysis. Abbreviations: GS, grower standard; B1, vitamin B1; Bc, vitamin B complex; BcE, vitamin B complex + vitamin E; BcCE, vitamin B complex + vitamin C + vitamin E. Suffixes -50 and -250 denote application concentration (µg mL^−1^).

The flower-to-nut conversion ratio was analysed for both growing seasons. As shown in Figure 7, there was no statistically significant difference between the untreated control and the grower standard and no significant difference between the grower standard and all vitamin treatments (B1-50 to BcCE-250).

The flower-to-nut conversion ratio varied across treatments, with considerable variability observed within several treatment groups. Among the vitamin-based treatments, Bc-50 showed the highest median flower-to-nut conversion ratio, followed by BcCE-250 and B1-250, with values generally higher than the untreated control. By contrast, B1-50, BcE-50, BcCE-50, and Bc-250 showed comparatively lower median conversion ratios. BcE-250 also demonstrated a relatively high conversion ratio, although substantial variation was observed among samples. Overall, the results indicate that flower-to-nut conversion differed among vitamin formulations and application concentrations; however, these observed numerical differences should be interpreted alongside the corresponding statistical significance analysis.

### 3.5. Leaf Chlorophyll Level Assessments

To determine whether the vitamin treatments negatively impacted chlorophyll production in the plants, SPAD chlorophyll values were checked over 1920 individual leaves grouped into 96 different samples, each consisting of 20 leaves. Statistical tests were conducted to understand this impact at a 95% confidence level.

According to the statistical analysis, there is no significant difference between the control and treated trees in terms of chlorophyll levels (*p* = 0.996) and in Season 2 (*p* = 0.527). However, we observed that there is a significant difference in chlorophyll levels among the different varieties, and this phenomenon was consistent across both growing seasons. 

As discussed in the results for both growing seasons, there was no significant difference between the control and the treatments. Per the statistical analysis, Season 1 resulted in *p* = 0.99 and Season 2 resulted in *p* = 0.53. Chlorophyll was measured to ensure that there were no unintended physiological effects from the treatments. We can therefore conclude that the treatments had no negative impact on chlorophyll production in the plants.

### 3.6. Kernel Nutrient Assessments

**Figure 8:**
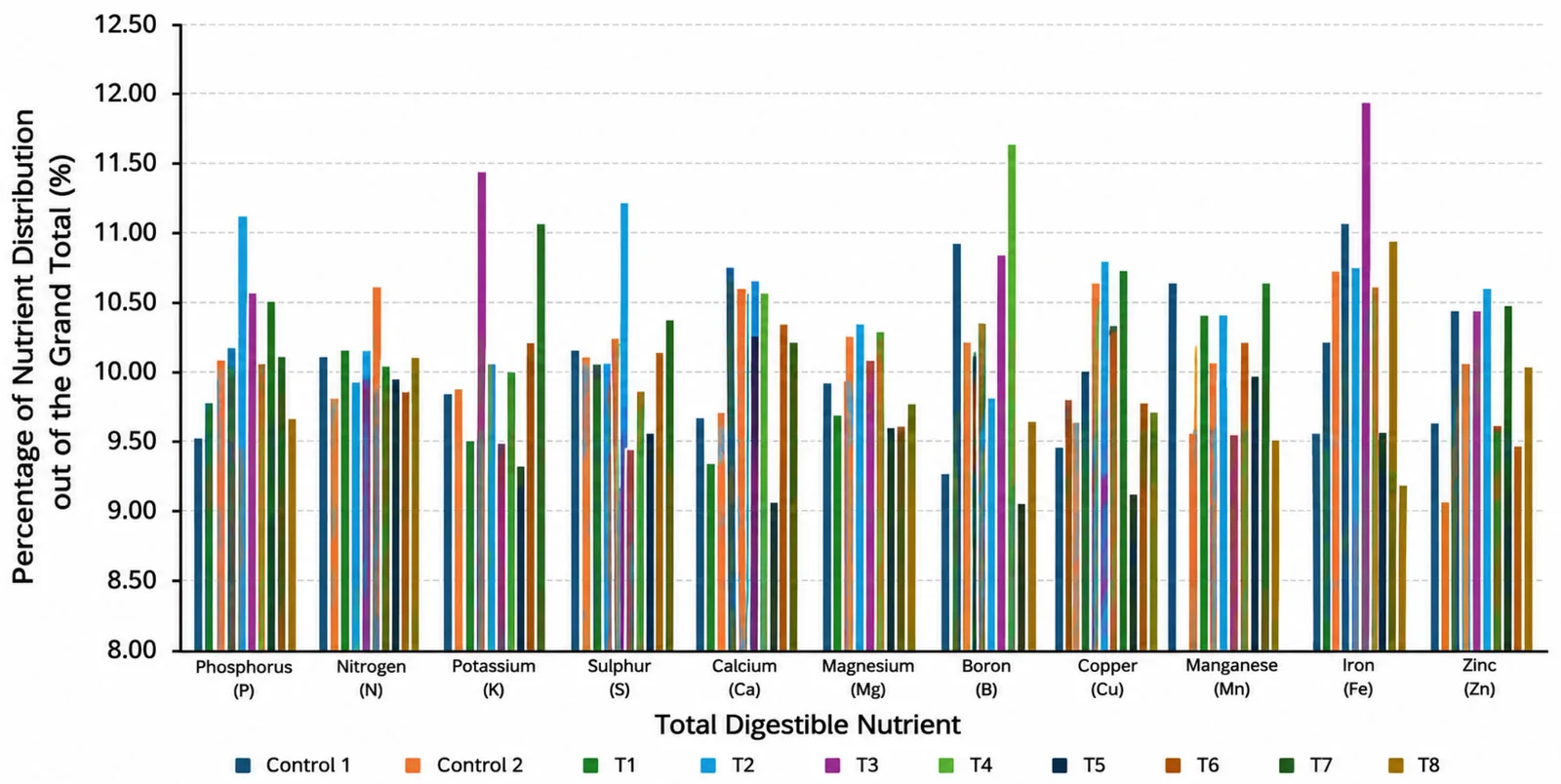
Nutrient distribution (as percentage) across Controls 1 and 2 and the eight vitamin treatments. (Abbreviations: GS, grower standard; B1, vitamin B1; Bc, vitamin B complex; BcE, vitamin B complex + vitamin E; BcCE, vitamin B complex + vitamin C + vitamin E. Suffixes -50 and -250 denote application concentration (µg mL^−1^). (B1-50) = T1, (Bc-50) = T2, (BcE-50) = T3, (BcCE-50) = T4, (B1-250) = T5, (Bc-250) = T6, (BcE-250) = T7, (BcCE-250) = T8. Control 1 = no treatments, Control 2 = GS).

To visualise the distribution of nutrients, each nutrient value is represented in Figure 8 as a segment of its grand total. The analysis shows that all 11 nutrients are almost equally distributed between 8% and 12%. There was no statistically significant difference in the 95% confidence level between the control (Control) and grower standard (GS) Control 2 or between the B1-50(T1) and BcCE-250 (T8) treatments.

The relative distribution of total digestible nutrients varied moderately among the treatments, with values generally ranging from approximately 9% to 12% of the grand total. Phosphorus, potassium, sulphur, calcium, copper, magnesium, iron, and zinc showed relatively comparable distributions across most treatments, although several treatment-specific variations were evident. The most pronounced responses were observed for potassium, calcium, and iron, where individual treatments produced noticeably higher nutrient distributions than the respective controls. In particular, the highest overall response was observed for iron, reaching approximately 12%, while elevated values were also recorded for calcium (approximately 11.6%) and potassium (approximately 11.4%). Despite these individual peaks, the overall distribution remained relatively consistent across nutrients, which suggests that the vitamin-based treatments influenced the relative nutrient profile selectively rather than producing a uniform increase across all measured nutrients.

### 3.7. Almond Hulls and Kernel Maximum Residue Level (MRL) Assessments

Because reducing pesticide dependence and residue risk in almond hulls was a central sustainability objective of this study, a multi-residue pesticide screen was conducted on hull and kernel samples from both growing seasons. A total of 280 analytes were included, comprising organochlorine and organophosphate pesticides, fungicides, insecticides, carbamates, synthetic pyrethroids, neonicotinoids and chloroacetamide herbicides. Residues were detected primarily in samples from the grower standard treatment (Table 10), whereas the vitamin-based programme was evaluated as a strategy to reduce reliance on these conventional pesticide inputs. No elevated pesticide residues were detected in almond kernels in either growing season.

These results demonstrate the practical relevance of residue management when almond hulls are intended for livestock feed or other value-added uses. Although most detected residues in the grower standard samples were below the applicable limits, the occurrence of measurable residues illustrates the potential for pesticide use in the orchard to carry through to the hull by-product stream. This residue assessment therefore complements the disease control results by evaluating whether a lower-pesticide disease management strategy could support safer and more sustainable hull utilisation.

### 3.8. Almond Kernel Yield Assessment

As kernel weight is the most important aspect of almond production, it is essential to ensure that any novel treatment does not negatively impact the weight of the kernels. A reduction in kernel weight would mean that the novel treatment was an impractical solution to the challenge of reducing chemical pesticide usage.

According to the statistical analysis of the growing Season 1 results (as in summary Table A5 and Table A6), the oven-dried almond kernels from the treated plants and controls show no statistical difference in Season 1 (*p* = 0.18) or in Season 2 (*p* = 0.21).

However, according to the statistical analysis summary in Table A7 and Table A8 (in the Appendix A section), there is a significant difference in kernel weight among the varieties. This is the expected outcome, as kernel weight and sizes vary according to the varieties.

The statistical analysis of oven-dried kernel weight showed no significant treatment effect in either growing season (Season 1, *p* = 0.181; Season 2, *p* = 0.211). Thus, the vitamin treatments had no measurable effect on kernel yield relative to the untreated control or grower standard. By contrast, selected vitamin formulations reduced pathogen-associated disease incidence while maintaining the measured kernel-quality attributes. These responses may also support post-harvest quality by reducing pathogen pressure, although storage stability was not measured directly in this study. Importantly, this disease management response was achieved with a reduced reliance on conventional pesticide applications and, consequently, a lower risk of pesticide residues entering the almond hull by-product stream.

### 3.9. Almond Kernel Damage Caused by Insects, Including the Carob Moth and Carpophilus Beetle

Kernel damage attributed to carob moth and Carpophilus beetle was generally low across both growing seasons, which indicates that pest incidence was limited throughout the study period (Table A9 and Table A10).

In Season 1, only a single damaged kernel was recorded across all treatments and almond varieties. This damage occurred in the untreated control within the Nonpareil variety, representing 100% of the total recorded kernel damage for the season. No kernel damage was observed in the Carmel or Monterey varieties, and no damage was detected in any of the treatment groups, including the grower standard and treatments B1-50–BcCE-250. Overall, kernel damage during the first season was negligible.

In Season 2, a greater number of damaged kernels was observed, with a total of 19 kernels affected across all treatments. Damage remained absent in the Carmel variety, while Monterey recorded three damaged kernels, all occurring in the untreated control. The majority of the damage was observed in the Nonpareil variety, where 16 damaged kernels (84.21% of the seasonal total) were recorded. Among the treatments, BcE-50 accounted for the highest number of damaged kernels, with 10 kernels (52.63% of the seasonal total), followed by the untreated control with five kernels (26.32%). Treatments B1-250 and BcCE-250 each recorded two damaged kernels (10.53%), whereas the grower standard and treatments B1-50, Bc-50, BcCE-50, Bc-250, and BcE-250 showed no detectable kernel damage.

Across both seasons, kernel damage remained infrequent, with only one damaged kernel recorded in Season 1 and 19 damaged kernels in Season 2. The distribution of damage was concentrated predominantly in the Nonpareil variety, whereas Carmel showed no evidence of kernel damage in either season. Several treatments, including the grower standard, consistently recorded no detectable kernel damage throughout this study, while differences in damage incidence among the remaining treatments were relatively small because of the low overall number of affected kernels.

## 4. Discussion

As shown in Figure 2 and Figure 3 above, B1-50 (vitamin B1-50 µg mL^−1^) and BcE-50 (vitamin B complex + vitamin E-50 µg mL^−1^) demonstrated a relatively higher efficacy in suppressing disease pressure. Meanwhile, BcE-50 (vitamin B complex + vitamin E-50 µg mL^−1^) and BcCE-50 (vitamin B complex + vitamin C + vitamin E-50 µg mL^−1^) exhibited remarkable control when considering the control of whole-nut disease, while B1-250 also showed higher control within the vitamin treatment group. BcE-50 and BcCE-50 were the top performers with respect to kernel insect damage suppression [28].

Identifying the commonalities across these treatments and understanding the logical mechanisms behind their effectiveness is essential. The concentration of 50 µg mL^−1^ proved to be effective in controlling leaf damage. The primary observation indicates that, in many instances, lower vitamin concentrations were more effective than higher concentrations. B1-50, BcE-50 and BcCE-50 utilised a 50 µg mL^−1^ vitamin B complex formula, and it was observed that this formulation was more effective at lower concentrations, although vitamin B1 remained effective at a concentration of 5x. These observations are in line with the results of a number of studies conducted in the area of human health [29] that have shown that the consumption of vitamin B helps repel insect bites. We observed that this phenomenon was applicable to almond crops as well. The observed decrease in pest and disease occurrence can therefore be seen to confirm findings from prior studies. Specifically, vitamin B complex functions as a plant defence activator, enhancing systemic acquired resistance (SAR), priming the plant’s defences, and inhibiting the growth of pathogens [30]. This process activates pathogenesis-related (PR) gene expression, enhances protein kinase C activity, and triggers PR-1 activation. As a result, the plant has a quicker and stronger response to infection [31]. Further, vitamin B1 (thiamine) promotes the synthesis of beneficial compounds, such as salicylic acid (SA) and phenolic compounds, which play a significant role in enhancing overall plant health and increasing disease resistance.

Although vitamin C is a well-known antioxidant, its role in plant defence extends beyond the simple scavenging of hydrogen peroxide. During pathogen attack, plants generate a rapid and transient oxidative burst that functions as a signalling mechanism to activate defence pathways. Vitamin C contributes to maintaining cellular redox homeostasis by preventing excessive accumulation of reactive oxygen species after defence signalling has been initiated, thereby protecting host tissues from oxidative damage while preserving normal physiological function. Likewise, vitamin E protects cellular membranes against lipid peroxidation caused by oxidative stress, helping maintain membrane stability during defence responses. Consequently, these antioxidants are considered to complement rather than suppress ROS-mediated defence signalling and may contribute to improved disease tolerance by enhancing the plant’s ability to withstand oxidative stress associated with pathogen infection.

The reduction in disease incidence without a corresponding increase in kernel yield requires careful interpretation. Kernel yield did not differ significantly among treatments in Season 1 (*p* = 0.181) or Season 2 (*p* = 0.211), despite significant treatment effects on leaf and whole-nut disease incidence. Under the commercial orchard conditions of this study, disease pressure was measurable but remained sufficiently controlled, such that it did not translate into a detectable yield penalty. Accordingly, the results should not be interpreted as evidence that disease is unrelated to almond productivity, nor that the vitamin treatments suppressed yield. Rather, the vitamin treatments reduced disease while maintaining a kernel yield comparable with those of the untreated control and grower standard. Trials conducted under greater disease pressure, across additional sites and seasons, will be required to determine whether the observed disease suppression can produce a measurable yield advantage.

Further research is needed to understand the impact of a first growing season on the second growing season. In our case, we observed that both leaf disease levels and overall plant disease levels were lower in Season 2. This reduction may be attributed to seasonal effects or the influence of treatments administered during Season 1, which may have inhibited the occurrence of pests and diseases in the subsequent season. In both growing seasons, the whole-nut disease control treatments were observed to have had more than double the disease occurrence compared to the grower standard or any of the vitamin formulation-treated samples. This suggests that the grower standard practices effectively controlled the diseases investigated in this study. As there was no significant difference in the disease control levels for B1-50 to BcCE-250 (vitamin B complex + vitamin C + vitamin E-250 µg mL^−1^), we have reason to believe that the vitamin formulas were effective in controlling whole-nut disease levels. A minor deviation was nonetheless observed between Season 1 and Season 2 as we were attempting to determine the most effective treatment within the group. BcE-50 and BcCE-50 were most frequently found to have the most effective formulations.

While the analysis of the flower-to-nut conversion ratios revealed no significant difference between the untreated control and the standard chemical application used by growers, the untreated control resulted in over 15% more nuts than the grower standard. This observation may be explained by the negative interactions between the chemical pest control agents and bee pollination. Further, Bc-50 (vitamin B complex-50 µg mL^−1^) demonstrated the highest flower-to-nut conversion ratio, which may have been due to the successful and higher rate of bee pollination. Prior research has shown that including B vitamins in bee diets notably impacts their overall carbohydrate intake [32]. We propose, therefore, that the specific vitamin treatment in Bc-50 stimulated the bees’ need for higher carbohydrates, and the bees consequently actively visited a greater number of almond flowers, which resulted in an increased level of successful pollination.

Careful examination of the nutrient distribution percentage across the 10 different treatments (as illustrated in Figure 8) led to several important observations that may inform future research directions and provide opportunities for manipulating the nutrient composition of almond kernels to enhance their quality. Notably, the levels of zinc, iron, copper, magnesium, nitrogen, phosphorus and sulphur were significantly elevated in kernels derived from the BcCE-50-treated almond trees. This phenomenon may be explained by findings from previous studies that indicate that certain organic acids, such as vitamin C (ascorbic acid), play a pivotal role in improving the absorption of iron and zinc from available sources by forming soluble chelates with these minerals. We have reason to believe that the foliar allocation of BcCE-50 (vitamin B complex + vitamin C + vitamin E-50 µg mL^−1^), which consists of vitamin C, interacted with recent foliar nutrient applications, which led to effective uptake and subsequently higher concentrations in the kernels. Even though this was not statistically significant, we suggest that this could be the focus of future studies.

The MRL analysis indicated that the almond hulls contained elevated levels of Azoxystrobin. While this strobilurin fungicide is commonly used, it comes with certain health concerns, especially regarding developmental toxicity and neurotoxicity [29]. Although its potential for acute toxicity is low, studies have shown that it may disrupt mitochondrial functions [30], leading to decreased energy production, which could hinder neuronal development and functionality. Furthermore, research points to Azoxystrobin exposure potentially increasing levels of reactive oxygen species (ROS), which can induce oxidative stress and affect oocyte maturation [31]. Additionally, some studies suggest that Azoxystrobin may encourage cell death and that it exhibits neurotoxic effects [30] at concentrations relevant to human exposure. The second highest residue found in the almond hulls was Methoxyfenozide insecticide. Studies have shown that Methoxyfenozide can cause mild blood thinning and liver abnormalities [32], an observation that further strengthens the argument that the almond industry should search for a suitable and effective substitute for chemicals that elevate MRL risk.

From an economic perspective, the vitamin-based treatments evaluated in this study are commercially attractive because the cost of the vitamin applications was estimated to be approximately five times lower than that of conventional chemical pesticide applications used for disease management in commercial almond production. When combined with the observed reduction in disease incidence and the potential to reduce pesticide residues in almond hulls, these lower application costs improve the commercial viability of vitamin-based biostimulants as a sustainable component of integrated disease management. Nevertheless, future studies should include a comprehensive cost–benefit analysis incorporating treatment costs, disease control efficacy, yield, and long-term economic returns under commercial orchard conditions.

## 5. Conclusions

This study evaluated the performance of several vitamin-based biostimulant formulations under commercial almond orchard conditions over two consecutive growing seasons. Selected vitamin treatments were consistently associated with lower disease incidence in both leaves and whole nuts compared with untreated trees.

Although several vitamin formulations significantly reduced disease incidence, they did not produce a statistically significant change in kernel yield over the two growing seasons. Instead, the treatments maintained yields at levels comparable with the untreated control and the grower standard while preserving flower-to-nut conversion, chlorophyll status and the measured kernel nutrient composition. Selected formulations therefore provided a quality-focused benefit through lower pathogen-associated disease pressure, without compromising crop productivity. This reduction in pathogen pressure may also favour post-harvest quality and storage stability; however, storage life was not evaluated directly, and this potential benefit requires confirmation in dedicated post-harvest studies. From a sustainability perspective, reducing reliance on conventional pesticide applications also lowers the risk of pesticide residues in almond hulls intended for livestock feed or other value-added uses.

Several treatments performed at a level comparable to the grower standard chemical programme, which suggests that vitamin-based biostimulants may provide a practical option for disease management in commercial almond production.

A particular strength of this work is that it was conducted under commercial orchard conditions, which allowed the treatments to be evaluated under the environmental and management conditions experienced by growers. In addition to disease suppression, this study considered crop performance, kernel quality and pesticide residues, providing a broader assessment of the practical value of vitamin-based biostimulants than has previously been reported for almond production.

The reductions in disease observed in this study are consistent with earlier reports that vitamins can contribute to improved plant health and tolerance to biotic stress. However, the present work focused on field performance rather than the physiological or molecular processes underlying these responses. Consequently, the mechanisms responsible for the observed effects were not investigated directly and should not be inferred from the present results alone. Further research combining field experiments with physiological, biochemical and molecular analyses would provide a better understanding of how vitamin-based biostimulants influence disease development in almond trees.

From a practical perspective, the findings have implications beyond disease management. Reducing reliance on conventional pesticide applications may assist growers in producing almond hulls with lower pesticide residue risks, thereby improving opportunities for their use as livestock feed and other value-added products. Improving the utilisation of almond by-products would increase resource efficiency, reduce waste and support more sustainable almond production systems.

This study also has some limitations that should be recognised. This research was conducted at a single commercial orchard in one almond-growing region of Australia and evaluated a defined range of vitamin formulations over two growing seasons. While these conditions reflect commercial production practices, further studies across different production regions, seasonal conditions and orchard management systems are needed before broader recommendations can be made. Future research should also investigate application timing, application frequency, optimum rates and the long-term economic performance of vitamin-based biostimulants under commercial production conditions.

Overall, the results indicate that vitamin-based biostimulants have the potential to become a useful component of integrated disease management in commercial almond production. While additional research is required across a wider range of environments and disease pressures, the consistent responses observed over two consecutive seasons show that selected vitamin formulations can reduce disease pressure while maintaining crop performance. In practical terms, the treatments did not increase total kernel yield, but they maintained measured kernel quality and may offer post-harvest benefits through lower pathogen pressure while reducing dependence on pesticides and the associated residue risk. These findings provide a practical foundation for developing more sustainable disease management strategies for the almond industry.

## Figures and Tables

**Table 1:** Application dosages of different vitamin treatments and short notations. The abbreviations defined in Table 1 are used consistently in the Results, figures and Discussion: B1, vitamin B1 (thiamine); Bc, commercial vitamin B complex; BcE, vitamin B complex + vitamin E; and BcCE, vitamin B complex + vitamin C + vitamin E.

Short Notation	Concentration	Treatments
(B1-50) T1	50 µg mL^−1^	Vitamin B1
(Bc-50) T2	50 µg mL^−1^	Vitamin B complex
(BcE-50) T3	50 µg mL^−1^	Vitamin B complex + E (1:1)
(BcCE-50) T4	50 µg mL^−1^	Vitamin B complex + C + E (1:1:1)
(B1-250) T5	250 µg mL^−1^	Vitamin B1
(Bc-250) T6	250 µg mL^−1^	Vitamin B complex
(BcE-250) T7	250 µg mL^−1^	Vitamin B complex + E (1:1)
(BcCE-250) T8	250 µg mL^−1^	Vitamin B complex + C + E (1:1:1)
Control 1	No chemical or vitamin treatments	Control (no chemical or vitamin treatments)
Control 2	Grower standard chemical application	Grower standard (grower standard chemical application)

**Table 2:** Impact of Leaf Disease Damage in Season One at the Varietal Level.

Season 1	Carmel		Monterey		Nonpareil		All Varieties	
Treatments	Mean of Leaf Damage	Mean as a Percentage	Mean of Leaf Damage	Mean as a Percentage	Mean of Leaf Damage	Mean as a Percentage	Sum of Means of Leaf Damage	Sum of Means as a Percentage
**Control**	26	7.01%	26	7.01%	23	6.20%	75	20.22%
**Grower standard**	12	3.23%	8	2.16%	10	2.70%	30	8.09%
**B1-50**	15	4.04%	4	1.08%	7	1.89%	26	7.01%
**Bc-50**	10	2.70%	10	2.70%	14	3.77%	34	9.16%
**BcE-50**	6	1.62%	14	3.77%	16	4.31%	36	9.70%
**BcCE-50**	7	1.89%	6	1.62%	13	3.50%	26	7.01%
**B1-250**	18	4.85%	9	2.43%	16	4.31%	43	11.59%
**Bc-250**	9	2.43%	14	3.77%	15	4.04%	38	10.24%
**BcE-250**	13	3.50%	13	3.50%	7	1.89%	33	8.89%
**BcCE-250**	9	2.43%	7	1.89%	14	3.77%	30	8.09%
**Grand total**	125	33.69%	111	29.92%	135	36.39%	371	100.00%

**Table 3:** Impact of Leaf Disease Damage in Season Two at the Varietal Level.

Season 2	Carmel		Monterey		Nonpareil		All Varieties	
Treatments	Mean of Leaf Damage	Mean as a Percentage	Mean of Leaf Damage	Mean as a Percentage	Mean of Leaf Damage	Mean as a Percentage	Sum of Means of Leaf Damage	Sum of Means as a Percentage
**Control**	12	10.17%	12	10.17%	10	8.47%	34	28.81%
**Grower standard**	6	5.08%	3	2.54%	5	4.24%	14	11.86%
**B1-50**	0	0.00%	0	0.00%	0	0.00%	0	0.00%
**Bc-50**	7	5.93%	5	4.24%	3	2.54%	15	12.71%
**BcE-50**	7	5.93%	2	1.69%	1	0.85%	10	8.47%
**BcCE-50**	3	2.54%	4	3.39%	1	0.85%	8	6.78%
**B1-250**	3	2.54%	2	1.69%	3	2.54%	8	6.78%
**Bc-250**	2	1.69%	6	5.08%	2	1.69%	10	8.47%
**BcE-250**	3	2.54%	3	2.54%	3	2.54%	9	7.63%
**BcCE-250**	2	1.69%	6	5.08%	2	1.69%	10	8.47%
**Grand total**	45	38.14%	43	36.44%	30	25.42%	118	100.00%

**Table 4:** Impact of Whole-Nut Disease Damage in Season One at the Varietal Level.

Season 1	Carmel		Monterey		Nonpareil		All Varieties	
Treatments	Mean of Nut Damage	Mean as a Percentage	Mean of Nut Damage	Mean as a Percentage	Mean of Nut Damage	Mean as a Percentage	Sum of Means of Nut Damage	Sum of Means as a Percentage
**Control**	44	7.61%	48	8.30%	50	8.65%	142	24.57%
**Grower standard**	20	3.46%	14	2.42%	8	1.38%	42	7.27%
**B1-50**	25	4.33%	21	3.63%	15	2.60%	61	10.55%
**Bc-50**	28	4.84%	19	3.29%	8	1.38%	55	9.52%
**BcE-50**	21	3.63%	25	4.33%	5	0.87%	51	8.82%
**BcCE-50**	19	3.29%	7	1.21%	12	2.08%	38	6.57%
**B1-250**	20	3.46%	19	3.29%	3	0.52%	42	7.27%
**Bc-250**	28	4.84%	20	3.46%	5	0.87%	53	9.17%
**BcE-250**	24	4.15%	21	3.63%	6	1.04%	51	8.82%
**BcCE-250**	22	3.81%	19	3.29%	2	0.35%	43	7.44%
**Grand total**	251	43.43%	213	36.85%	114	19.72%	578	100.00%

**Table 5:** Impact of Whole-Nut Disease Damage in Season Two at the Varietal Level.

Season 2	Carmel		Monterey		Nonpareil		All Varieties	
Treatments	Mean of Nut Damage	Mean as a Percentage	Mean of Nut Damage	Mean as a Percentage	Mean of Nut Damage	Mean as a Percentage	Sum of Means of Nut Damage	Sum of Means as a Percentage
Control	40	8.75%	41	8.97%	55	12.04%	136	29.76%
Grower standard	4	0.88%	1	0.22%	48	10.50%	53	11.60%
**B1-50**	10	2.19%	17	3.72%	25	5.47%	52	11.38%
**Bc-50**	9	1.97%	1	0.22%	25	5.47%	35	7.66%
**BcE-50**	5	1.09%	0	0.00%	0	0.00%	5	1.09%
**BcCE-50**	8	1.75%	8	1.75%	16	3.50%	32	7.00%
**B1-250**	10	2.19%	0	0.00%	3	0.66%	13	2.84%
**Bc-250**	8	1.75%	1	0.22%	17	3.72%	26	5.69%
**BcE-250**	7	1.53%	22	4.81%	31	6.78%	60	13.13%
**BcCE-250**	10	2.19%	18	3.94%	17	3.72%	45	9.85%
Grand total	111	24.29%	109	23.85%	237	51.86%	457	100.00%

**Table 6:** Almond Kernel Damage Due to Carob Moth and Carpophilus Beetle in Season One at the Varietal Level.

Season 1	Carmel		Monterey		Nonpareil		All Varieties	
Treatments	Total Kernel Damage	Total as Percentage	Total Kernel Damage	Total as Percentage	Total Kernel Damage	Total as Percentage	Sum of Total Kernel Damage	Sum of Total as Percentage
**Control**	0	0.00%	0	0.00%	1	100.00%	1	100.00%
**Grower standard**	0	0.00%	0	0.00%	0	0.00%	0	0.00%
**B1-50**	0	0.00%	0	0.00%	0	0.00%	0	0.00%
**Bc-50**	0	0.00%	0	0.00%	0	0.00%	0	0.00%
**BcE-50**	0	0.00%	0	0.00%	0	0.00%	0	0.00%
**BcCE-50**	0	0.00%	0	0.00%	0	0.00%	0	0.00%
**B1-250**	0	0.00%	0	0.00%	0	0.00%	0	0.00%
**Bc-250**	0	0.00%	0	0.00%	0	0.00%	0	0.00%
**BcE-250**	0	0.00%	0	0.00%	0	0.00%	0	0.00%
**BcCE-250**	0	0.00%	0	0.00%	0	0.00%	0	0.00%
**Grand total**	0	0.00%	0	0.00%	1	100.00%	1	100.00%

**Table 7:** Almond Kernel Damage Due to Carob Moth and Carpophilus Beetle in Season Two at the Varietal Level.

Season 2	Carmel		Monterey		Nonpareil		All Varieties	
Treatments	Total Kernel Damage	Total as Percentage	Total Kernel Damage	Total as Percentage	Total Kernel Damage	Total as Percentage	Sum of Total Kernel Damage	Sum of Total as Percentage
**Control**	0	0.00%	3	15.79%	2	10.53%	5	26.32%
**Grower standard**	0	0.00%	0	0.00%	0	0.00%	0	0.00%
**B1-50**	0	0.00%	0	0.00%	0	0.00%	0	0.00%
**Bc-50**	0	0.00%	0	0.00%	0	0.00%	0	0.00%
**BcE-50**	0	0.00%	0	0.00%	10	52.63%	10	52.63%
**BcCE-50**	0	0.00%	0	0.00%	0	0.00%	0	0.00%
**B1-250**	0	0.00%	0	0.00%	2	10.53%	2	10.53%
**Bc-250**	0	0.00%	0	0.00%	0	0.00%	0	0.00%
**BcE-250**	0	0.00%	0	0.00%	0	0.00%	0	0.00%
**BcCE-250**	0	0.00%	0	0.00%	2	10.53%	2	10.53%
**Grand total**	0	0.00%	3	15.79%	16	84.21%	19	100.00%

**Table 8:** Almond Kernel Damage Due to Aborting and Gumming in Season One at the Varietal Level.

Season 1	Carmel		Monterey		Nonpareil		All Varieties	
Treatments	Total Kernel Damage	Total as Percentage	Total Kernel Damage	Total as Percentage	Total Kernel Damage	Total as Percentage	Sum of Total Kernel Damage	Sum of Total as Percentage
**Control**	0	0.00%	0	0.00%	0	0.00%	0	0.00%
**Grower standard**	1	12.50%	0	0.00%	0	0.00%	1	12.50%
**B1-50**	0	0.00%	0	0.00%	0	0.00%	0	0.00%
**Bc-50**	0	0.00%	0	0.00%	1	12.50%	1	12.50%
**BcE-50**	0	0.00%	0	0.00%	1	12.50%	1	12.50%
**BcCE-50**	1	12.50%	0	0.00%	2	25.00%	3	37.50%
**B1-250**	0	0.00%	1	12.50%	0	0.00%	1	12.50%
**Bc-250**	0	0.00%	0	0.00%	0	0.00%	0	0.00%
**BcE-250**	0	0.00%	1	12.50%	0	0.00%	1	12.50%
**BcCE-250**	0	0.00%	0	0.00%	0	0.00%	0	0.00%
**Grand total**	2	25.00%	2	25.00%	4	50.00%	8	100.00%

**Table 9:** Almond Kernel Damage Due to Aborting and Gumming in Season Two at the Varietal Level.

Season 2	Carmel		Monterey		Nonpareil		All Varieties	
Treatments	Total Kernel Damage	Total as Percentage	Total Kernel Damage	Total as Percentage	Total Kernel Damage	Total as Percentage	Sum of Total Kernel Damage	Sum of Total as Percentage
**Control**	1	3.23%	1	3.23%	0	0.00%	2	6.45%
**Grower standard**	0	0.00%	1	3.23%	2	6.45%	3	9.68%
**B1-50**	3	9.68%	2	6.45%	1	3.23%	6	19.35%
**Bc-50**	7	22.58%	0	0.00%	0	0.00%	7	22.58%
**BcE-50**	0	0.00%	0	0.00%	2	6.45%	2	6.45%
**BcCE-50**	0	0.00%	2	6.45%	1	3.23%	3	9.68%
**B1-250**	0	0.00%	0	0.00%	0	0.00%	0	0.00%
**Bc-250**	0	0.00%	0	0.00%	0	0.00%	0	0.00%
**BcE-250**	0	0.00%	3	9.68%	3	9.68%	6	19.35%
**BcCE-250**	0	0.00%	1	3.23%	1	3.23%	2	6.45%
**Grand total**	11	35.48%	10	32.26%	10	32.26%	31	100.00%

**Table 10:** Summary of reported chemical residues in grower standard in Seasons 1 and 2.

Season 1				Season 2		
Pesticide Residues in mg/kg	Grower Standard Carmel	Grower Standard Monterey	Grower Standard Nonpareil	Grower Standard 2 Carmel	Grower Standard Monterey	Grower Standard Nonpareil
**Fungicides**						
Azoxystrobin *	0.03	<0.01	0.04	7.43	3.49	6.45
Propiconazole *	0.70	<0.01	1.14	<0.01	0.05	0.22
**Insecticides**						
Chlorantraniliprole *	3.72	0.07	2.52	<0.01	<0.01	<0.01
Methoxyfenozide *	2.94	<0.01	3.65	2.94	<0.01	3.65
**Neonicotinamides**						
Clothianidin *	<0.01	<0.01	0.11	<0.01	<0.01	<0.01

## Data Availability

The data supporting the findings of this study are contained within the article. Additional data and supporting materials are available from the corresponding author upon reasonable request.

## Appendix A

Detailed statistical output tables and data for parameters without significant treatment effects are provided in this Appendix A, while the principal treatment-level disease data are presented in the main Results.

**Table A1:** Summary of Type III Tests of Fixed Effects from the Linear Mixed Model for Season One. F values and corresponding significance (*p*) values are presented.

Source	Numerator df	Denominator df	F	Sig.
Intercept	1	60.177	138.678	<0.001
Variety	2	60.177	0.444	0.643
Treatment	9	59.967	5.717	<0.001
Variety * Treatment	18	59.967	0.550	0.920

Dependent variable is total disease-damaged sites per leaf.

**Table A2:** Summary of Type III Tests of Fixed Effects from the Linear Mixed Model for Season Two. F values and corresponding significance (*p*) values are presented.

Source	Numerator df	Denominator df	F	Sig.
Intercept	1	60.177	28.724	<0.001
Variety	2	60.177	0.696	0.502
Treatment	9	59.967	5.199	<0.001
Variety * Treatment	18	59.967	0.675	0.821

Dependent variable is total disease-damaged sites per leaf.

**Table A3:** Season one: Pairwise comparisons, Control 1 vs the other nine treatments. Std. error denotes standard error; df denotes degree of freedom; and sig. denotes significant at 95% confidence level. To be statistically significant, the sig. value should be lower than 0.05. The dependent variable is the leaf chlorophyll levels, based on estimated marginal means. The Bonferroni method was used to adjust for multiple comparisons.

(I) Treatment	(J) Treatment	Mean Difference (I-J)	Std. Error	df	Sig.	95% Confidence Interval for Difference	
						Lower Bound	Upper Bound
Control 1	Grower standard	−0.483	1.341	52.110	1.000	−5.114	4.148
	T1	−0.517	1.372	57.165	1.000	−5.228	4.194
	T2	−0.383	1.372	57.165	1.000	−5.094	4.328
	T3	−0.594	1.372	57.165	1.000	−5.306	4.117
	T4	0.083	1.372	57.165	1.000	−4.628	4.794
	T5	−1.039	1.372	57.165	1.000	−5.750	3.672
	T6	0.039	1.372	57.165	1.000	−4.672	4.750
	T7	−0.939	1.372	57.165	1.000	−5.650	3.772
	T8	−0.028	1.372	57.165	1.000	−4.739	4.683

**Table A4:** Season two: Pairwise comparisons, Control 1 vs the other nine treatments. Std. error denotes standard error; df denotes degree of freedom; and sig. denotes significant at 95% confidence level. To be statistically significant, the sig. value should be lower than 0.05. The dependent variable is the leaf chlorophyll levels, based on estimated marginal means. The Bonferroni method was used to adjust for multiple comparisons.

(I) Treatment	(J) Treatment	Mean Difference (I-J)	Std. Error	df	Sig.	95% Confidence Interval for Difference	
						Lower Bound	Upper Bound
Control 1	Grower standard	−0.392	1.388	52.110	1.000	−5.182	4.399
	T1	−0.306	1.419	57.165	1.000	−5.179	4.568
	T2	0.783	1.419	57.165	1.000	−4.090	5.657
	T3	−0.128	1.419	57.165	1.000	−5.001	4.746
	T4	−1.061	1.419	57.165	1.000	−5.935	3.813
	T5	1.806	1.419	57.165	1.000	−3.068	6.679
	T6	1.872	1.419	57.165	1.000	−3.001	6.746
	T7	0.572	1.419	57.165	1.000	−4.301	5.446
	T8	1.083	1.419	57.165	1.000	−3.790	5.957

**Table A5:** Summary Table of Average Kernel Weight Type III Tests of Fixed Effects in Growing Season One.

Source	Numerator df	Denominator df	F	Sig.
Intercept	1	60.223	1986.937	<0.001
Treatment	9	59.959	1.466	0.181
Variety	2	60.223	6.057	0.004
Treatment * Variety	18	59.959	0.456	0.967

Dependent variable: oven-dry kernel weight.

**Table A6:** Summary Table of Average Kernel Weight Type III Tests of Fixed Effects in Growing Season Two.

Source	Numerator df	Denominator df	F	Sig.
Intercept	1	60.223	1610.912	<0.001
Treatment	9	59.959	1.395	0.211
Variety	2	60.223	10.524	<0.001
Treatment * Variety	18	59.959	0.902	0.579

Dependent variable: oven-dry kernel weight.

**Table A7:** Season one: Pairwise comparisons of the untreated control vs the other nine treatments. The dependent variable was the oven-dried kernel weight, based on estimated marginal means. The Bonferroni method was used to adjust for multiple comparisons. Std. error denotes standard error; df denotes degree of freedom; and sig. denotes significance at the 95% confidence level. To be statistically significant, the sig. value should be lower than 0.05.

(I) Treatment	(J) Treatment	Mean Difference (I-J)	Std. Error	df	Sig.	95% Confidence Interval for Difference	
						Lower Bound	Upper Bound
Control 1	Grower Standard	0.116	2.122	52.110	1.000	−7.209	7.441
	T1	−3.040	2.170	57.165	1.000	−10.492	4.412
	T2	−3.637	2.170	57.165	1.000	−11.089	3.814
	T3	−4.217	2.170	57.165	1.000	−11.669	3.234
	T4	−4.956	2.170	57.165	1.000	−12.408	2.495
	T5	−3.174	2.170	57.165	1.000	−10.626	4.278
	T6	−2.170	2.170	57.165	1.000	−9.622	5.282
	T7	−2.681	2.170	57.165	1.000	−10.133	4.771
	T8	−5.380	2.170	57.165	0.726	−12.832	2.072

**Table A8:** Season two: Pairwise comparisons of untreated control vs other nine treatments. The dependent variable was the oven-dried kernel weight, based on estimated marginal means. The Bonferroni method was used to adjust for multiple comparisons. Std. error denotes standard error; df denotes degree of freedom; and sig. denotes significance at the 95% confidence level. To be statistically significant, the sig. value should be lower than 0.05.

(I) Treatment	(J) Treatment	Mean Difference (I-J)	Std. Error	df	Sig.	95% Confidence Interval for Difference	
						Lower Bound	Upper Bound
Control 1	Grower standard	−4.359	2.403	52.110	1.000	−12.655	3.936
	T1	0.326	2.457	57.165	1.000	−8.113	8.765
	T2	−2.099	2.457	57.165	1.000	−10.538	6.340
	T3	0.214	2.457	57.165	1.000	−8.225	8.653
	T4	−0.451	2.457	57.165	1.000	−8.890	7.988
	T5	0.999	2.457	57.165	1.000	−7.440	9.438
	T6	3.384	2.457	57.165	1.000	−5.055	11.823
	T7	−1.570	2.457	57.165	1.000	−10.009	6.869
	T8	−1.396	2.457	57.165	1.000	−9.835	7.043

**Table A9:** Almond Kernel Damage Due to Carob Moth and Carpophilus Beetle in Season One at the Varietal Level.

Season 1	Carmel		Monterey		Nonpareil		All Varieties	
Treatments	Total Kernel Damage	Total as Percentage	Total Kernel Damage	Total as Percentage	Total Kernel Damage	Total as Percentage	Sum of Total Kernel Damage	Sum of Total as Percentage
**Control**	0	0.00%	0	0.00%	1	100.00%	1	100.00%
**Grower standard**	0	0.00%	0	0.00%	0	0.00%	0	0.00%
**T1**	0	0.00%	0	0.00%	0	0.00%	0	0.00%
**T2**	0	0.00%	0	0.00%	0	0.00%	0	0.00%
**T3**	0	0.00%	0	0.00%	0	0.00%	0	0.00%
**T4**	0	0.00%	0	0.00%	0	0.00%	0	0.00%
**T5**	0	0.00%	0	0.00%	0	0.00%	0	0.00%
**T6**	0	0.00%	0	0.00%	0	0.00%	0	0.00%
**T7**	0	0.00%	0	0.00%	0	0.00%	0	0.00%
**T8**	0	0.00%	0	0.00%	0	0.00%	0	0.00%
**Grand total**	0	0.00%	0	0.00%	1	100.00%	1	100.00%

**Table A10:** Almond Kernel Damage Due to Carob Moth and Carpophilus Beetle in Season Two at the Varietal Level.

Season 2	Carmel		Monterey		Nonpareil		All Varieties	
Treatments	Total Kernel Damage	Total as Percentage	Total Kernel Damage	Total as Percentage	Total Kernel Damage	Total as Percentage	Sum of Total Kernel Damage	Sum of Total as Percentage
**Control**	0	0.00%	3	15.79%	2	10.53%	5	26.32%
**Grower standard**	0	0.00%	0	0.00%	0	0.00%	0	0.00%
**T1**	0	0.00%	0	0.00%	0	0.00%	0	0.00%
**T2**	0	0.00%	0	0.00%	0	0.00%	0	0.00%
**T3**	0	0.00%	0	0.00%	10	52.63%	10	52.63%
**T4**	0	0.00%	0	0.00%	0	0.00%	0	0.00%
**T5**	0	0.00%	0	0.00%	2	10.53%	2	10.53%
**T6**	0	0.00%	0	0.00%	0	0.00%	0	0.00%
**T7**	0	0.00%	0	0.00%	0	0.00%	0	0.00%
**T8**	0	0.00%	0	0.00%	2	10.53%	2	10.53%
**Grand total**	0	0.00%	3	15.79%	16	84.21%	19	100.00%
